# Dual circulation strategy, regional healthcare development, and medical collaborative innovation efficiency: evidence from Chinese cities

**DOI:** 10.3389/fpubh.2024.1371867

**Published:** 2024-04-26

**Authors:** Lixia Chen, Jianyuan Huang, XinYan Ge

**Affiliations:** ^1^School of Marxism, Hefei Normal University, Hefei, China; ^2^School of Public Administration, Hohai University, Nanjing, China; ^3^Nanjing Vocational University of Industry Technology, Nanjing, China

**Keywords:** dual circulation strategy, medical collaborative innovation efficiency, threshold test, regional healthcare development, China

## Abstract

This study analyzes panel data of Chinese cities from 2003 to 2018 as a sample in the context of the dual circulation strategy in China to ascertain the impact of urban healthcare development on medical collaborative innovation efficiency by using the GS2SLS method. Furthermore, it empirically examines the influence mechanism of regional healthcare development on medical collaborative innovation efficiency by using a threshold regression model. Additionally, we identified the heterogeneity of this impact in different cities. The results show the following: (1) There is a significant positive spatial correlation between regional healthcare development and medical collaborative innovation efficiency; (2) Under the dual circulation strategy, the regional investment level in international circulation has the most significant role in the overall strategy, and domestic circulation has been significantly improved after the launch of the innovation-driven strategy; (3) The results of the threshold test show that while domestic and international circulation promote the efficiency of collaborative innovation by 0.83, the promotion effect is more obvious under a higher regional healthcare development level. The research in this paper can provide specific guidance for the development of China’s healthcare industry under the background of dual-cycle strategy, and can also provide valuable reference for developing countries in the world.

## 1 Introduction

China proposed in 2020 to develop a new development pattern with the domestic cycle as the main body, where the domestic and international dual circulation strategies promote each other. A country cannot achieve high-quality development without developing its own domestic economic development cycle or an external international economic development cycle. The cycle of domestic economic development in the country, especially the process of globalization over the past century, has increased the impact of the cycle of international economic development. Driven by the industrial revolution, and scientific and technological progress, a large number of commodities took advantage of their comparative advantages to flock to the international market; thereby promoting globalization and the development of the world economy. Economic globalization accelerated again in the early years of the State, following the weakening of the two world wars, restrictions on immigration, and the Great Depression, especially after many developing countries joined the international economic cycle in the 1980s. Since the reform and opening up of its economy, China has actively integrated into the world market to participate in the international cycle. The upgradation of the industrial structure and the development of technological innovation has systematically improved China’s position and voice in the global value chain.

However, in the face of the outbreak of COVID-19, some shortcomings in the construction and development of China’s medical system have gradually emerged, such as insufficient reserves of special drugs, high drug prices, and relatively backward medical science and technology. The high-quality development of the medical and health industry is an important guarantee for the safety of people’s lives. It is an important goal of health economics to improve the level of medical science and technology innovation and realize the optimal allocation of medical resources under the condition of resource constraints. Therefore, in the context of the new dual-cycle development pattern, how to achieve high-quality development of medical care and improve the level of regional medical collaborative innovation is of great significance to achieve high-level development.

At present, although a large number of research literatures have carried out in-depth analysis of the double-cycle strategy, there are few literatures that specifically examine the development of healthcare and the collaborative innovation of inter-regional medical industry under the background of the double-cycle strategy. At the same time, most of the existing research literature is based on linear regression model, ignoring the inter-regional collaborative innovation and spatial spillover effect, and to some extent ignoring the inter-regional linkage effect. This not only has a bias in the estimation results of empirical research, but also cannot provide very accurate policy recommendations.

This paper thus constructs an analytical framework, and explores domestic and international dual circulation based on the impact of domestic and international dual circulation strategies on the medical collaborative innovation efficiency by combining it with the level of regional healthcare development. It achieves this by taking 283 cities at the prefecture level and above in China from 2003 to 2018 as a sample, and the efficiency of urban industry-university-research medical collaborative innovation as the explanatory variable. The different impact effects and degrees of the development level of each specific link of the strategy under the threshold of the healthcare development level of different regions are analyzed to enrich and deepen the current research on healthcare innovation and development under the new development pattern, and provide useful enlightenment and policy suggestions for cities in different regions at different stages of development to better achieve medical innovative development.

## 2 Literature review and theoretical analysis

### 2.1 Literature review

The literature on the dual circulation strategy mainly focuses on the elaboration of the theoretical level, which is an objective need that has not changed in the last 100 years (1) and has many decisive factors (2). China already has a strong production and supply capacity, and the data released by the National Bureau of Statistics in January 2020 show that the average gross domestic product (GDP) of China has exceeded 10,000 US dollars, and has begun to demonstrate the demand constraint characteristics of the mature market economy. Thus, demand-side reform from the perspective of the dual circulation strategy is of great significance. Understanding the dual circulation strategy from the perspective of political economy focuses on opening up the production, circulation, consumption, and distribution links between domestic and foreign countries (3). In addition, some studies have provided relevant explanations for the dual circulation strategy from the perspective of empirical analysis at the macro level: Ding et al. examined the internal and external orientation choices of China’s economic cycle from 1987 to 2017 by constructing an interprovincial transfer and export comparison preference index and found that China as a whole is currently dominated by the domestic large cycle (4); similarly, Yang studied the structural changes of China’s economy from 1994 to 2019 from the perspective of the development pattern of the dual circulation strategy by constructing and measuring the indicator system reflecting the economic development situation (5). The theoretical analysis framework of domestic and international dual circulation strategies is constructed using input–output models that reveal the current situation and characteristics of the strategies. Accordingly, it can be seen that China’s economic development has characteristics exhibited by domestic circulation (6).

With the advent of the Internet era, new generation information technologies such as big data and cloud computing have had a profound impact on the development of the medical industry. Stelzner et al. believe that with the continuous development of nanotechnology, medical technologies that cannot be realized due to the lack of the original refinement level can be realized under nano conditions, and it has a wide range of potential applications in medical communication (7). Dananjayan et al. believes that with the popularization and application of 5G technology, its characteristics across time and space can enable patients to realize “face to face” communication with doctors without the cost of long-distance commuting. Virtual consultation and remote consultation are becoming a new development direction of medical technology, and therefore play an irreplaceable role in the regional medical level (8). Wang et al. believe that supervised and unsupervised learning based on machine learning methods and data obtained from clinical or actual investigations can effectively improve the accuracy and scientific nature of disease prediction, construct intelligent medical scenarios, and realize effective application in practice (9).

The improvement of medical collaborative innovation efficiency is a process of exchange, collaboration, and collaborative innovation between various entities such as enterprises, colleges and universities, scientific research institutions, and government departments within the system. The collaborative utilization of resources and the joint development of technology are also realized in the region. Compared with the evaluation of innovation efficiency, the analytical perspective of collaborative innovation considers the correlation effect between various subjects within the innovation system and between various regional systems, which is more beneficial for achieving an innovation system. Current research on collaborative innovation mainly focuses on technological innovation, innovation efficiency, innovation ecosystems, innovation networks, and industry-university research; the methods used in collaborative innovation research in recent years are mainly system dynamics, evolutionary games, social network analysis, and other methods (10). Experts and scholars have conducted a lot of research on the regional collaborative innovation; specifically, scholars mainly from the connotation of collaborative innovation (11), the model of collaborative innovation (12), the mechanism of collaborative innovation (13), and other aspects of theoretical discussion (14) have also empirically examined the impact of the linkage relationship between enterprises, universities, research institutes, governments, and financial institutions within the regional innovation system and the efficiency of regional innovation.

Several relevant studies have been conducted in the academic community regarding the relationship between regional healthcare development and innovation. Most studies take innovation as the starting point and the core research area, to sort out its role in promoting regional healthcare development (15). Simultaneously, some studies believe that the relationship between innovation and regional healthcare development is more complicated, and their interaction and mutual influence form a coupled developmental relationship of mutual promotion and harmonious symbiosis (16). Scholars have studied the role of regional healthcare development in promoting innovation in terms of providing a good innovation environment (17), attracting talent (18), and providing better material conditions and financial input for innovative activities (19).

Thus, it can be surmised that the existing literature has carried out relevant research and analysis on the pattern of dual circulation strategy and medical collaborative innovation efficiency from the theoretical and empirical aspects. This provides an important reference for the research of this paper. However, the evaluation and measurement of regional dual circulation strategy are more effective, and the empirical analysis of the combination of strategy and medical collaborative innovation efficiency needs to be improved and expanded further.

### 2.2 Theory development

As a development deployment in the new era, the scientific connotation of the new development pattern lies in internal circulation as the mainstay, and external circulation as a supplement. From the perspective of the new development pattern, this study uses the framework of regional domestic and international economic cycles to analyze and discuss the impact of regional healthcare development on the efficiency of collaborative innovation.

The ideological origin of the new development pattern comes from the Marxist theory of the political economy. As Marx states in Capital, the expansion of reproduction of the entire society of production, distribution, circulation, and consumption of the capitalist economy is based on the domestic circulation of the capitalist economy. The production process of the capitalist economy is essentially the production of surplus value, but Marx’s socialized large-scale production theory, based on the capitalist economy, applies to the analysis of the operation and circulation of the socialist market economy with Chinese characteristics as well. Therefore, using these concepts and logic to analyze the laws of the modern market economy, including the cycle of the socialist market economy, is also completely in line with Marxist positions and viewpoints (20).

Based on the above theoretical analysis, the essence of the national economic cycle is the social reproduction process, and the social reproduction theory is the basic tool for analyzing the domestic large cycle and the international economic cycle (21). Thus, there is a cyclical relationship between production, distribution, circulation, and consumption. Since these four processes cover most aspects of economic and social life, it provides a unified framework for the analysis of the economy and society. Therefore, the development of regional economies can be evaluated and interpreted from the perspective of the two major economic cycles in China and globally. Based on a collation and analysis of the existing literature, we believe that the specific links of the domestic and international dual circulation strategy can have an impact on the efficiency of regional innovation at the relevant level.

The improvement in the level of regional healthcare development is manifested in the production link as the improvement of the overall production capacity of the region, and production, as the starting point and foundation of social and economic activities. The improvement of its ability can naturally provide guarantee and support for the improvement of regional innovation efficiency. Specifically, the improvement of regional production levels can mainly affect regional innovation efficiency through the spillover effect of technical space (22), the improvement of the comprehensive support capacity of various undertakings and the progress of the management level and related systems (14).

In terms of distribution, the development of the regional economy is reflected in the more equal distribution of regional income, the structure and method of income distribution are more reasonable, and unequal income distribution is controlled and improved. The improvement in the income distribution pattern is also relatively clear for the improvement of regional innovation efficiency (23). On the demand side, unequal income distribution will significantly dampen consumer demand for innovative products, and thereby reduce domestic innovation and R&D investment (24). From an input perspective, maintaining the income gap at a high level, will reduce the total investment in research and development of the country, and damage its ability for independent innovation. While modest levels of inequality in income distribution can contribute to an increase in innovation levels, there is a consistent correlation between income distribution levels and regional innovation efficiency in the long run.

In terms of circulation, the development of the regional economy will inevitably be accompanied by the improvement of regional infrastructure and transportation accessibility. The developed regional commercial trade network and circulation system have also played an irreplaceable and important role in the improvement of regional innovation efficiency. Furthermore, developing the circulation industry can also effectively strengthen the region’s independent innovation ability (25, 26).

Consumption, as the final link in the large-scale production of the domestic economic society, plays an important role in the field of economic development, and the vitality of regional consumption is also an important embodiment and evaluation indicator of the degree of development of the regional economy. The improvement in regional spending power can also promote innovation efficiency in the region in various ways. At the micro level, it will lead users to discover new needs in the market (27). Once the business adopts these market needs, it is applied to R&D and production, and then it will reap greater innovation performance. The development process of capitalist developed economies confirms the important role played by consumption levels and structures on the demand side in stimulating technological innovation and progress at the macro level (28).

Since joining the WTO in 2001, as an important member of the world economic system, China has actively participated in international trade and the international economic cycle and has established extremely close ties with the global economic system through decades of reform. Overall, a country’s participation in the international economic cycle is mainly through import and export trade, foreign direct investment (FDI), and outward direct investment (OFDI). The impact of participation in the international economic cycle on innovation mainly comes from international technology spillover, and the main channels of international technology spillover are FDI and international import and export trade. Scholars have conducted a large amount of relevant research on theoretical mechanisms and empirical data focusing on the impact of FDI and import and export trade on innovation (29–31). Among them, FDI can influence the innovation of the host country through spillover and thus form two opposing hypotheses: the polluted paradise hypothesis and the pollution aura hypothesis (32). The former suggests that the transfer of low-end industries from developed countries to developing countries, and the locking of developing countries at the lower end of the value chain leads to the inhibition of innovation in developing countries. The latter suggests that developed countries will bring advanced technologies to promote innovation in developing countries. The ultimate effect depends on which effect is stronger, as both occur simultaneously. In addition, some scholars have studied the impact of international trade on innovation and found that the development of international trade plays a significant role in promoting and enhancing China’s technological innovation ability.

Based on the above research and analysis, it can be observed that scholars have systematically analyzed and studied the impact of innovation and collaborative innovation from the perspectives of different parts of the domestic economic cycle and the international economic cycle. However, the empirical research on the systematic analysis of the impact mechanism of collaborative innovation from the overall perspective of the new development pattern has not yet been explored. In the era of global integration, any economy in the process of development has both a domestic cycle and a foreign cycle of two-cycle systems, the domestic market and the world market you have me, I have you, and the economic ties between the two are inseparable. Participating in the domestic and international economic cycles is mutually superficial, constituting a system of joint promotion of economic development. Therefore, it comprehensively reflects the specific level of regional economy under the current development pattern. Thus, this study considers the analysis framework of constructing a domestic and international economic cycle based on the new development pattern and studies its correlation with the efficiency of collaborative innovation.

## 3 Model and variables

### 3.1 Basic model setup and construction

The empirical part of this study focuses on identifying the impact of domestic and international dual circulation strategies on the efficiency of collaborative innovation. That is, the mechanism and intensity of the development level of various economic links in the city on the efficiency of collaborative innovation. The basic regression model was set as follows in Equation (1):


(1) $$Y_{it}=\alpha_{0}+\alpha_{1}E_{it}+\alpha_{2}X_{it}+\alpha_{3}C_{it}+\varepsilon_{it}$$

Among them, the explanatory variable *Yit* is the medical collaborative innovation efficiency of prefecture-level city i in *year t*, and the core explanatory variable is the economic development level of prefecture-level city i in year t. Eit *Xit* A is a set of variables that reflect the regional economy, and the domestic and international circulation level, including the variables that reflect the relevant levels of production, distribution, circulation, and consumption of the regional domestic economic cycle level, the total FDI of the region, and the total imports and exports of the region. These reflect the level of the regional economic cycle. *Cit* was another control variable. Since the medical collaborative innovation efficiency of a city is not only related to the innovation resources and environment of the city itself but is also affected by the spillover effect of the medical collaborative innovation efficiency of neighboring cities, the medical collaborative innovation efficiency of two cities that are geographically adjacent to each other will have a spatial correlation to a certain extent. Expanded model (1) introduces the effect of medical collaborative innovation efficiency in neighboring cities, and the expanded spatial regression model is Equation (2)


(2) $$Y_{it}=\alpha_{0}+\alpha_{1}\overset{n}{\underset{j=1}{\sum }}W_{ij}Y_{jt}+\alpha_{2}E_{it}+\alpha_{3}X_{it}+\alpha_{4}C_{it}+\varepsilon_{it}$$

$$\varepsilon_{it}=\lambda \overset{n}{\underset{j=1}{\sum }}w_{ij}\varepsilon_{it}+\mu_{it}$$

where *W* is the spatial weight matrix reflecting the connection of each city at the spatial level, and the element in the spatial weight matrix reflects the spatial connection between the cities. is the spatial lag coefficient and is the coefficient of spatial error, which reflects the spatial dependence of the sample observations and the spatial correlation that exists in the error structure, respectively. When the value is zero, the spatial econometric model is a spatial lag model; when the value is zero, the spatial econometric model is a spatial error model. The spatial lag coefficient and spatial error coefficient together reflect the interaction and effect of medical collaborative innovation efficiency between samples of neighboring cities, as well as their spatial spillover effects $w_{ij}\alpha_{1}\lambda \lambda \alpha_{1}$ .

### 3.2 Spatial weights matrix

In contrast to traditional econometric research, spatial econometric research focuses on the processing of spatial data, and the so-called spatial data adds the position information or mutual distance of the cross-sectional units to the original cross-sectional or panel data (33). Therefore, the spatial weight matrix used to measure the spatial distance between regions must be introduced into the econometric analysis. Therefore, this study selects a geographic distance weight matrix based on the interregional geographic distance factor, such that that *W* = *ω*, *where ω* represents the weight of the geographic distance spatial weight matrix, *and W* represents the geographic distance spatial weight matrix and its elements *_wij._* It is the reciprocal of the absolute difference in geographical distance between city i and city j. The geographic distance data between cities is the straight-line distance between cities, calculated based on the longitude and latitude data obtained from the National Basic Geographic Information System 1:4 million terrain database.

### 3.3 Variable description

#### 3.3.1 Dependent variables

Medical collaborative innovation efficiency (*innov*). The concept of “collaborative innovation” was first proposed by Gloor (34), and since then, researchers at home and abroad have conducted in-depth exploration and empirical analysis around its connotation, mode, mechanism, and effect evaluation. Based on the relevant research of Bai and Bian (35), the efficiency of collaborative innovation can be mainly divided into two types: intraregional and interregional. In this study, using the network DEA model, MaxDEA software was used to measure the performance of collaborative innovation in 283 cities from 2003 to 2018, and the average of their annual total efficiency was selected to represent the specific value of medical collaborative innovation efficiency.

Since collaborative innovation within the region involves many subjects and links, the research process in the innovation process will be ignored if the ordinary data envelopment analysis model is used, which can result in inaccurate calculation of the efficiency of collaborative innovation within cities. Through the network DEA theoretical system, the input and output in the innovation process can be quantified concretely, thus solving the problem of the input and output process as a “black box” and resulting in a lack of data in the intermediate links. The innovation triple spiral structure of enterprises, universities, and scientific research institutes in the city, and the interaction of innovative talents, capital, and other elements between various innovative subjects within it, are circular. The chain relationship between the innovative subjects can be divided into two stages: “learning to research” and “research to production.” The two stages form a circular structure through the connection between the innovation subjects. “Learning to research” refers to the process in which universities obtain funds through cooperation with enterprises or government transfer payments, and use them as capital to cultivate the innovative talents needed for the next stage of output. “Research to production” refers to the process by which scientific research institutions transform the funds obtained from enterprises and the government, and the innovative talents absorbed from universities into scientific research results and put them into production and application. This study refers to the input–output index selection system in the relevant research of Wang (36) to quantitatively analyze the innovation efficiency in accordance with the order of “learning to research” and “research to production,” decompose the cycle structure into a chain structure, and use the chain network DEA to quantify the medical collaborative innovation efficiency. Each city above the prefecture level is a decision unit DMUI (*i* = 1, 2, .., n), assuming that there are s (*s* = 1, 2, ...,) in the whole process *S* phase, the input variables and output variables of each stage are *_Iis_* and *_Ois_*, respectively, and satisfy *Ii ^s^*∈ *R_+_^αs^* and *_Ois_*∈ *R_+_^βs^*; the intermediate variables of the s and s + 1 phases are set to *Pi^(s,s + 1)^* and satisfy *Pi^(s,s + 1)^* ∈ *R_+_^γ (s, s + 1),^* where *α*, *β*, and *γ* represent the number of input variables, output variables, and intermediate variables, respectively. *α* = 1, 2, ..., *x*，*β* = 1, 2, ..., *y*，*γ* = 1, 2, ..., *z* 。 *λS* is the model weight, *wS* is the weight variable of the sth order throughout the process, and *λS* ∈ *R _+_^n^*, *μ ^s-^*, and *μ ^s+^* are the relaxation variables of the input and output variables, respectively. The target θ of the network envelope analysis model can be expressed as in Equations (3, 4):


(3) $$\begin{array}{l} \theta =\min\frac{\sum_{s=1}^{S}\omega^{s}[1-\frac{1}{\alpha }(\sum_{x=1}^{\alpha }\frac{\mu_{x}^{s-}}{I_{x_{0}}^{s}})]}{\sum_{s=1}^{S}\omega^{s}[1+\frac{1}{\beta }(\sum_{y=1}^{\beta }\frac{\mu_{x}^{s+}}{O_{y_{0}}^{s}})]}\\ \left\{ \begin{array}{l} I_{0}^{s}=\sum_{i=1}^{n}\lambda_{i}^{s}I_{i}^{s}+\mu^{s-}\\ O_{0}^{s}=\sum_{i=1}^{n}\lambda_{i}^{s}O_{i}^{s}+\mu^{s+}\\ P^{\left(s,s+1\right)}\lambda^{s+1}=P^{\left(s,s+1\right)}\lambda^{s}\\ \sum_{i=1}^{N}\lambda_{i}^{s}=\sum_{s=1}^{S}\omega^{s}=1\\ \lambda^{S},\mu^{s-},\mu^{s+},w^{S}\geq 0 \end{array}\right. \end{array}$$

(4) $$\theta_{s}=\frac{1-\frac{1}{\alpha }\left(\overset{\alpha }{\underset{x=1}{\sum }}\frac{\mu_{x}^{s-*}}{I_{x_{0}}^{s}}\right)}{1+\frac{1}{\beta }\left(\overset{\beta }{\underset{y=1}{\sum }}\frac{\mu_{x}^{s+*}}{O_{y_{0}}^{s}}\right)}$$

With reference to the data requirements of relevant research and network envelope analysis model, this paper selects the number of students in ordinary colleges and universities, the number of urban unit education practitioners and public finance education expenditure from the two aspects of human resources and capital to reflect the input of the first stage of “learning to research”; the number of scientific and technological practitioners of urban units is selected as the output of the first stage of learning and research. Then, the number of scientific and technological employees in urban units, public finance and scientific and technological expenditures, and the stock of fixed assets in the whole society are taken as the inputs for the second stage of research and production; the number of scientific research papers, patents, and GDP are used as the scientific research output and economic output of the second stage of “research and production.”

#### 3.3.2 Gravitational model

Since each region is not independent, the efficiency of collaborative innovation is affected not only by factors within the city but also by the flow of factors throughout the network of innovative cities. Collaborative innovation between industry, academia, and research also occurs in closed areas because of the flow and agglomeration of different types of innovation elements between cities. Therefore, it is necessary to measure the efficiency of collaborative innovation between regions from the spatial correlation perspective. The gravitational model is a successful application of the law of gravitation in physics to the social sciences and is mainly used to study the problem of spatial interaction in an economic society. Based on the gravitational model, medical collaborative innovation efficiency between cities is manifested as the collaborative development process of medical collaborative innovation efficiency within the city; whereby, there is an increase in the difficulty of absorption and spillover of innovation activities and a decrease in the collaborative innovation correlation between the two places when there is an increase in the economic gap between two places. For this reason, this study refers to the practice of Fan et al. (37) and others, constructs the following gravitational model from an economic point of view, and uses it to measure the efficiency of interregional collaborative innovation:


(5) $$Inter\_innov_{i}=\overset{n}{\underset{j=1}{\sum }}\frac{Intra\_innov_{i}\times Intra\_innov_{j}}{d_{ij}^{2}}$$

In Equation (5), that is, the performance of city $Inter\_innov_{i}$ *i* in the inter-city collaborative innovation under the gravitational model, and the product of the $Intra\_innov_{i}\times Intra\_innov_{j}$ collaborative innovation performance between city *i* and city *j*, the geographical distance between city $d_{ij}^{2}$ *i* and city *j*.

#### 3.3.3 Independent variables

According to the theoretical analysis above, the domestic economic circulation system can be divided into four main parts: production, distribution, circulation, and consumption. These four parts complement each other and are indispensable, forming a closely connected circulatory system. This study starts with these four links and selects representative indicators to measure the relevant performance of the region in terms of domestic circulation.

Production (production), is an important regional economic measurement index. The gross industrial output value above a designated size intuitively reflects the economic production scale and capacity of the region, which can effectively measure the overall economic output level of the region. Therefore, it is widely used in related studies to evaluate the productivity level of the region. Thus, this study uses the total industrial output value above the designated size to characterize the regional production capacity.

Income was measured by the ratio of rural to urban income in the region. Relevant studies have shown that China’s income gap is largely manifested as an urban–rural income gap. The level of the urban–rural income gap in the region is the distribution of output at the broadest level in the region, which reflects whether the output distribution in the region is reasonable (38). Referring to the relevant measurement methods of Xu et al. and Qian and Shen (39, 40), this study used the reciprocal ratio of *per capita* disposable income in urban areas to rural *per capita* net income to measure the relevant level of distribution in the region.

In terms of circulation (*transport*), modern innovation theory believes that innovation is the result of the interaction of various actors inside and outside the region (41); therefore, the selection of relevant indicators in circulation must reflect the mobility of production factors in the region. This was premised upon the relevant research by Tian et al. (42), who utilized the total amount of freight in the region to measure its circulation level.

In terms of consumption, the *per capita* consumption expenditure of the region, is the most intuitive embodiment of the consumption capacity of residents in the region, can be a good measure of the capacity and scale of the region in the field of consumption (43). Therefore, this study draws on the relevant research (44) and divides the total retail sales of social consumer goods by the total registered population of the region to obtain the *per capita* consumption expenditure of the region, and thereby measure the consumption level of the region.

Level of International Economic Circulation. Based on the above-mentioned research and theoretical analysis, the measurement and evaluation of the regional international economic cycle can be equivalent to the comprehensive systematic evaluation of the level of regional economic opening up. This study incorporates the level of attracting foreign investment (fdi) and regional import and export trade (trade) related to the international economic cycle, into the measurement analysis of the international economic cycle. This study selected the total annual FDI of the region and the total import and export trade of the region (45) as indicators to measure the level of attracting foreign investment (FDI) and the level of regional import and export trade (trade).

#### 3.3.4 Threshold variable

Regional healthcare development level (*rhd*). The impact of domestic and international economic cycles on different regions and different periods in the same region is not the same, and the threshold effect characteristics are formed based on the regional healthcare development level in the region because of the heterogeneity between the economic development level and innovation activities between different regions, and the heterogeneity between the regional healthcare development level and innovation activities in different periods of the same region. The level of economic development plays an important role in the process of opening up all aspects of production, distribution, circulation, and consumption and forming a new development pattern (1). Therefore, the construction of a new development pattern must be effectively connected with the regional development strategy and the level of regional healthcare development (46). This study thus takes the regional healthcare development level (*rhd*) as the threshold variable and explores whether the impact of large domestic and international cycles under the threshold of different regional healthcare development levels may be manifested as different degrees of promoting or inhibiting the efficiency of regional collaborative innovation. In previous related studies, GDP has generally been used in the evaluation of regional healthcare development levels, but since it has been used in DEA analysis, this study draws on relevant research to avoid endogenesis (47, 48), which uses the average brightness of lights at night to indicate the level of regional healthcare development. As an objective alternative economic indicator, night light data have a high positive correlation with GDP, and can accurately reveal the local economic development level.

#### 3.3.5 Control variables

This study refers to previous relevant studies on the efficiency of collaborative innovation at the city level (36) and controls some influencing factors related to innovation activities as control variables to join the model, including the total fixed asset investment (*fixed*) and electricity consumption in the region (*electric*) and regional industrial structure (*structure*) in terms of control variables to avoid the more serious bias of omission variables and to strip away the impact of regional healthcare development on the efficiency of urban collaborative innovation. The total investment in fixed assets in a region reflects the overall investment of the government in various construction projects in the region, and can effectively reflect the relevant level of infrastructure construction in the region. Electricity consumption represents the degree of economic vitality of manufacturing in a region, and the economic vitality of the manufacturing industry is inextricably linked to the innovation activities of the region (49). However, since most knowledge-intensive industries closely related to regional innovation belong to the tertiary industry, there should be a certain degree of correlation between the development and change of regional industrial structure and the efficiency of collaborative innovation (35). Therefore, this paper uses the proportion of tertiary industry output value as the tool of measurement. After controlling for the total amount of urban post and telecommunications business (*fixed*), electricity consumption (*electric*), and regional industrial structure (*structure*), it is more conducive to strip away the impact of regional healthcare development on the efficiency of urban collaborative innovation.

### 3.4 Data sources

The statistics used for the explanatory variables, explanatory variables, and control variables in this study are from the China Urban Statistical Yearbook (2004–2019) and the EPS database, and the parts with a small number of missing values are supplemented by linear smoothing. The number of scientific and technological papers used in the measurement of medical collaborative innovation efficiency consists of Chinese and English papers, Chinese papers, and English papers from CNKI and Web of Science (WOS) databases, and the number of three major patent applications (pieces) is retrieved from the “China Patent Full-text Database (CNKI Edition).” However, the variables used in the regression are logarithmic to eliminate the influence of heteroscedasticity on the study due to the existence of a comparative study of index mergers (Table 1).

**Table 1:** Descriptive statistics of variables.

Variable type	Index selection	Symbol	Index	Data source
Explained variable	medical collaborative innovation efficiency	innov	Input–output ratio of innovation behavior	It is calculated by DEA method
Core explanatory variable	regional healthcare development	rhd	Government medical expenses	Chinese urban Statistical Yearbook
Explanatory variable	Production level	produce	Gross industrial output value above designated size	Chinese urban Statistical Yearbook (2004–2019)
	Distribution level	income	The reciprocal ratio of urban per capita disposable income to rural per capita net income	
	Circulation level	transport	Regional freight volume	
	Consumption level	consume	Regional consumption expenditure per capita	
	Level of attracting foreign investment	fdi	Total annual foreign direct investment	EPS database
	International trade level	trade	Total import and export trade	
Control variables	Economic vitality of manufacturing industry	electric	Electricity consumption	Chinese urban Statistical Yearbook (2004–2019)
	infrastructure	fixed	Total investment in fixed assets	
	Industrial structure	structure	Proportion of output value of tertiary industry to total output value of regional industry	

## 4 Empirical results

### 4.1 Spatial correlation test of urban medical collaborative innovation efficiency

The Moran index reflects the degree of similarity in the values of the attributes of a spatially adjacent area cell, which is calculated as follows in Equation (6):


(6) $$\begin{array}{l} I=\frac{n\sum_{i=1}^{n}\sum_{j=1}^{n}\omega_{ij}\left(x_{i}-\bar{x}\right)\left(x_{j}-\bar{x}\right)}{\sum_{i=1}^{n}\sum_{j=1}^{n}\omega_{ij}\sum_{i=1}^{n}{\left(x_{i}-\bar{x}\right)}^{2}}\\ \;\;\;\;\;=\;\;\frac{\sum_{i=1}^{n}\sum_{j=1}^{n}\omega_{ij}\left(x_{i}-\bar{x}\right)\left(x_{j}-\bar{x}\right)}{S^{2}\sum_{i=1}^{n}\sum_{j=1}^{n}\omega_{ij}} \end{array}$$

n *_xi_* and *xj* represent the collaborative innovation efficiencies of city *i* and city *j*, respectively, and *S^2^* is the variance of *_i_* or *xj* $\bar{x}$ , which is the mean of *_xi_* or *xj*. ω *_ij_* is an element of the spatial weights matrix representing city *i* and city *j* neighbors. The Moran index has a range of values of [−1, 1]; positive numbers indicate the existence of a spatial positive correlation, negative numbers indicate the existence of a spatial negative correlation, and 0 indicates a random distribution characteristic of the space, i.e., there is no spatial correlation. Taking 283 cities at the prefecture level and above in China as the research object and medical collaborative innovation efficiency as the observation, the Moran index for 2003–2018 was calculated separately (see Table 2). The Moran index in different years passed the significance test, indicating that the medical collaborative innovation efficiency of cities at the prefecture level and above in China had a significant spatial autocorrelation.

**Table 2:** Moran-I index of collaborative innovation efficiency of 283 prefecture-level cities from 2003 to 2018.

Year	2003	2004	2005	2006	2007	2008	2009	2010
I	0.307	0.294	0.300	0.308	0.292	0.266	0.271	0.277
*p* value	0.000	0.000	0.000	0.000	0.000	0.000	0.000	0.000
Year	2011	2012	2013	2014	2015	2016	2017	2018
I	0.269	0.252	0.266	0.256	0.241	0.245	0.234	0.225
P value	0.000	0.000	0.000	0.000	0.003	0.030	0.008	0.096

### 4.2 Endogenous problems

A bidirectional causal relationship between the explanatory variables can lead to endogeneity problems. Schumpeter’s (50) theory of innovation and development regards innovation as the basic driving force of economic development. Thus, innovation efficiency can have an impact on economic development and economic development can provide a good material basis and environmental atmosphere for innovation, thereby promoting the development of innovation. Serious endogenous problems will make the least squares (OLS) estimation biased, and maximum likelihood estimation will fail when heteroscedasticity problems are present. Thus, the lag term of the explanatory variable can be selected as a tool variable to solve the problem of invalid estimation - the estimation is performed using the 2SLS method. However, considering the spatial spillover effect of innovation, we further selected the GS2SLS estimation, which selects each explanatory variable and its spatial lag term as the tool variable, and estimates the spatial panel model based on the 2SLS method while controlling the spatial correlation effects and endogenous problems in the model. The highest third-order spatial hysteresis term is selected as the tool variable for the datum regression, and the highest second-order spatial hysteresis term is selected as the tool variable for robustness testing.

### 4.3 Baseline regression analysis

Table 3 shows the GS2SLS estimates for the baseline model, and columns (1) and (2) are fixed-effects and random-effects model estimates that consider only the core explanatory variables and the model’s fundamental variables. Columns (3) and (4) add other control variables to columns (1) and (2), respectively. Columns (1), (2), (3), and (4) of Table 3 all pass the 1% significance level, indicating that a fixed-effects model should be chosen. The coefficients of the spatial lag terms of medical collaborative innovation efficiency in Table 3 are significantly positive at the 1% level, indicating that collaborative innovation has a significant spatial spillover effect and that the flow and transfer of innovation elements between regions can improve the medical collaborative innovation efficiency of neighboring regions.

**Table 3:** GS2SLS regression results.

Explanatory variable	Geographical distance Spatial Weight Matrix (W1)			
	(1)	(2)	(3)	(4)
	FE	RE	FE	RE
W_1_*lninnov	0.150***	0.029***	0.135***	0.014***
	(0.037)	(0.003)	(0.035)	(0.005)
lnproduce	0.001*	0.003***	0.001*	0.002***
	(0.001)	(0.001)	(0.001)	(0.001)
lnincome	0.009***	0.005***	0.004***	0.002***
	(0.001)	(0.001)	(0.001)	(0.001)
lntransport	0.002***	0.001**	0.001	0.003***
	(0.001)	(0.001)	(0.001)	(0.001)
lnconsume	0.009***	0.012***	0.006***	0.007***
	(0.001)	(0.001)	(0.001)	(0.001)
lnfdi	0.013***	0.014***	0.012***	0.013***
	(0.000)	(0.000)	(0.000)	(0.000)
lntrade	0.002***	0.004***	0.001***	0.004***
	(0.001)	(0.001)	(0.000)	(0.001)
Lnrhd	0.002***	0.005***	0.002***	0.005***
	(0.001)	(0.001)	(0.001)	(0.001)
lnelectric			0.003***	0.004***
			(0.001)	(0.001)
lnfixed			0.005***	0.004***
			(0.001)	(0.001)
lnstructure			0.006***	0.007***
			(0.001)	(0.001)
Adjusted R^2^	0.638	0.638	0.698	0.698
Wald test(p)	5416.211	5674.801	6341.145	6777.920
	(0.000)	(0.000)	(0.000)	(0.000)
Hausman test(p)	263.843 (0.000)		268.534 (0.000)	

***, ** and * represent significance levels of 1, 5 and 10% respectively. The value in square brackets below the coefficient is standard error; FE and RE represent fixed effects model and random effects model, respectively.

The influence of the interpreted variables representing the levels of various links in domestic and international economic cycles differs in terms of significance level and coefficient size. Specifically, among the variables representing the relevant levels of the four aspects of the regional economy’s domestic cycle, the regional consumption level has the greatest effect on improving collaborative innovation performance. Consumption, as the final link of the economic cycle, is one of the troikas that drives the macroeconomy alongside investment and exports, and its important role in economic growth has been widely recognized (51, 52). Similarly, it also plays a very important role in promoting regional innovation, and this role is mainly reflected through feedback from a backward nature (53). In his theory of consumption, Marx affirmed the role of consumption in promoting social production, economy, and innovation. The continuous development of society has led to an increase in consumption demand. Therefore, enterprises must improve efficiency, optimize processes, and develop new products to meet people’s increasing needs to survive in the face of fierce competition (54), which in turn will force all market participants to carry out more innovative activities. Among the variables representing the correlation level of the two aspects of the international cycle of the regional economy, the volume of FDI, and import and export trade have a significant positive impact on the efficiency of collaborative innovation, among which the positive impact of FDI on the efficiency of collaborative innovation is not only greater than the volume of import and export trade, but also greater than the explanatory variables representing the relevant level of the four aspects of the regional economy’s domestic cycle. FDI mainly affects regional innovation through technology spillovers, indicating that city-level technology spillovers play a vital role in improving innovation efficiency.

### 4.4 Robustness test

The robustness test of the benchmark regression results is mainly performed in this study by replacing the interpreted variables, spatial weights matrix, and instrumental variables. This is done by replacing the geographic distance spatial weight matrix (*W1*) previously used in regression with a nested weight matrix of geographic and economic distance (*W2*). Based on GS2SLS regression, the highest second-order spatial hysteresis term was used to replace the highest third-order spatial hysteresis term used in the previous regression as a tool variable. Table 4 shows the regression results, where the spatial lag term is still significant and the positive relationship between the core explanatory variables and the efficiency of collaborative innovation is still significant. The results show that the benchmark regression in the preceding study has strong robustness.

**Table 4:** Robustness test results.

Variable	Replacement is replaced by explanatory variable	Replacement space weight matrix	Replacement of instrument variable
W_1_*innov	2.437*** (0.164)	1.248*** (0.090)	1.205*** (0.021)
lnproduce	0.002** (0.001)	0.001* (0.001)	0.002*** (0.001)
lnincome	0.001 (0.001)	0.003** (0.001)	0.002** (0.001)
lntransport	0.004*** (0.001)	0.001* (0.001)	0.003*** (0.001)
lnconsume	0.004*** (0.001)	0.003** (0.001)	0.008*** (0.001)
lnfdi	0.012*** (0.000)	0.012*** (0.000)	0.013*** (0.000)
lntrade	0.001*** (0.001)	0.001* (0.000)	0.004*** (0.001)
lnrhd	0.003*** (0.001)	0.003*** (0.001)	0.002*** (0.001)
lnelectric	0.001 (0.001)	0.001 (0.001)	0.004*** (0.001)
lnfixed	0.000 (0.001)	0.002** (0.001)	0.004*** (0.001)
lnstructure	0.005*** (0.001)	0.004*** (0.001)	0.007*** (0.001)
Adjusted R^2^	0.810	0.508	0.739
Wald test(p)	490.614 (0.000)	6204.111 (0.000)	6779.203 (0.000)

W represents W_1_ in the replace interpreted variable, replace tool variable method, and W represents W_2_ in the replace space weight matrix method.

### 4.5 Regional heterogeneity test

According to the gradient development theory of regional economics, China’s economic regions are vertically divided into three major regions: the east, middle, and west (55)^1^. Since there are large economic development differences between these three regions, the 283 cities in the sample are divided into east, middle, and west according to the economic zones to which they belong, and spatial regression is carried out separately to explore whether there is a spatial spillover effect of regional healthcare development on the efficiency of collaborative innovation; the regression results are shown in Table 5. Comparing the regression results of various regions, we find that the role of distribution and consumption level in improving the efficiency of collaborative innovation is significant in the three regions with respect to the four aspects of the domestic economic cycle; this is consistent with the conclusions reached by the benchmark regression results. Specifically, the improvement of distribution and consumption levels plays the most obvious role in improving the efficiency of collaborative innovation in the central region, followed by the eastern region, and finally the western region. From the perspective of economic development, owing to the early opening up of the eastern region and the high level of economic development, the collaborative innovation of industry, education, and research has formed a relatively mature model, with the most significant spatial spillover effect. The central region is in an important stage of transformation and upgrading; therefore, the demand for innovation in all aspects of domestic economic development is more urgent, and the role of domestic economic development in promoting innovation is more obvious. However, because most western cities are limited by a weak foundation of local economic development and are late in opening up to the outside world, the level of economic development has lagged behind that of the central and eastern regions. In recent years, the western region has benefited from the internal transfer of industries supported by the policy of large-scale development in the western region. The western region has thus taken a leading position in the country in terms of economic development speed in recent years. However, due to the late development of the western region, the relevant supporting facilities and mechanisms still need to be improved, and the level of economic marketization is insufficient. Thus, the inequality of income distribution and the lack of consumption vitality have a greater restrictive effect on regional innovation.

**Table 5:** Regression by region.

Explanatory variable	Geographical distance spatial Weight Matrix (W_1_)		
	Eastern region	Central region	Western region
W_1_*lninnov	4.069*** (0.648)	1.203*** (0.277)	3.898*** (0.642)
lnproduce	0.001 (0.001)	0.001 (0.001)	0.001 (0.001)
lnincome	0.002** (0.001)	0.004*** (0.001)	0.001* (0.000)
lntransport	0.001 (0.001)	0.001 (0.001)	0.001 (0.001)
lnconsume	0.004*** (0.001)	0.007*** (0.002)	0.003*** (0.001)
lnfdi	0.011*** (0.001)	0.010*** (0.001)	0.011*** (0.001)
lntrade	0.002** (0.001)	0.001 (0.001)	0.001 (0.001)
lnrhd	0.003*** (0.001)	0.014*** (0.002)	0.022*** (0.002)
lnelectric	0.000 (0.000)	0.001 (0.001)	0.002** (0.001)
lnfixed	0.003*** (0.001)	0.001 (0.001)	0.001 (0.001)
lnstructure	0.001 (0.001)	0.003* (0.002)	0.003* (0.002)

***, ** and * represent significance levels of 1, 5 and 10% respectively; The values in square brackets below the coefficient are standard errors.

Among the two aspects of the international economic cycle, attracting foreign investment has maintained a positive and significant state in the three regions in improving the efficiency of collaborative innovation, and its coefficients are significantly greater than the four aspects of the domestic economic cycle. From the perspective of innovation, the regression results prove that there is an obvious positive spillover effect of FDI on the improvement of the efficiency of China’s industry-university-research collaborative innovation and affirm the role of foreign investment in promoting China’s innovative development.

### 4.6 Different stage test

Through the division of the overall period and the comparative changes, combined with the relevant theories, the phased characteristics of regional healthcare development for the performance improvement of collaborative innovation can be better analyzed and verified. The Eighteenth National Congress of the Communist Party of China held in 2012 clearly pointed out that scientific and technological innovation is a strategic support for improving social productivity and comprehensive national strength, and must be placed at the core of the overall development of the country. The Congress emphasized “that we must adhere to the path of independent innovation with Chinese characteristics and implement the innovation-driven development strategy.” The significance of the innovation-driven development strategy is to clarify that the direction of China’s future economic development will change from traditional labor and resource-driven model to an innovation-driven model, and that innovation must better serve development and economic construction. Under the guidance of the innovation-driven development strategy, China’s innovation industry has reached new heights, and the development of China’s economy is gradually transforming and upgrading in the direction of high quality and sustainability under the impetus of innovation. Therefore, this study takes 2012 as an important node of regional collaborative innovation and development, and divides 2003–2018 into two time periods: 2003–2012 and 2013–2018 for comparative analysis of benchmark regression. The regression results are presented in Table 6.

**Table 6:** Results of regression by stages.

Explanatory variable	Geographical distance spatial Weight Matrix (W_1_)	
	2003–2012	2013–2018
W_1_*lninnov	1.199***	2.167***
	(0.120)	(0.182)
lnproduce	0.001 (0.001)	0.001 (0.001)
lnincome	0.002** (0.001)	0.004*** (0.001)
lntransport	0.001 (0.001)	0.001 (0.001)
lnconsume	0.003*** (0.001)	0.007*** (0.002)
lnfdi	0.011*** (0.001)	0.007*** (0.001)
lntrade	0.002** (0.001)	0.001* (0.001)
lnrhd	0.003*** (0.001)	0.006*** (0.001)
lnelectric	0.000 (0.000)	0.001 (0.001)
lnfixed	0.003*** (0.001)	0.001 (0.001)
lnstructure	0.001 (0.001)	0.003* (0.002)

***, ** and * represent significance levels of 1, 5 and 10%, respectively.

The coefficient of the spatial lag term of medical collaborative innovation efficiency in Table 6 was significantly positive at the 1% level in both the 2003–2012 and 2013–2018 time periods, of which the coefficient of 2013–2018 was significantly greater than that of 2003–2012, indicating that the spatial spillover effect of medical collaborative innovation efficiency showed a gradually increasing trend. In terms of specific dual circulation strategy links, the coefficients of consumption level and distribution level in 2013–2018 increased significantly compared to 2003–2012, while the coefficient of foreign investment attraction and international trade level in 2013–2018 decreased compared to 2003–2012. This shows that in terms of promoting the efficiency of collaborative innovation, the various links in the circulation of the regional economy have gradually begun to play a more important role following the introduction of the innovation-driven strategy.

### 4.7 Threshold effect test

We will use the threshold regression model to conduct further research here to further explore the impact of domestic and international large cycles on the efficiency of collaborative innovation at different levels of economic development in different regions. “Threshold regression” tests for significant differences in the parameters of a sample group divided according to the threshold value. The threshold regression model developed by Hansen (56) can intrinsically divide data intervals according to the characteristics of the data themselves, avoiding the arbitrariness of artificially dividing the sample intervals. Therefore, this study adopts Hansen’s threshold regression model, takes the economic development level as the threshold variable, and combines the logarithmic form in the benchmark model to set the following single-threshold regression model in Equation (7):


(7) $$\begin{array}{c} \begin{array}{l} \ln innov_{it}=\alpha X_{it}+\beta l1nrhd_{it}\times I\left(\ln rhd_{it}\leq \gamma_{1}\right)\\ \;\;\;\;\;\;\;\;\;\;\;\;\;\;\;\;\;\;\;\;\;\;\;+\;\beta l2nrhd_{it}\times I\left(\gamma_{1}<\ln rhc_{it}\leq \gamma_{2}\right)\\ \;\;\;\;\;\;\;\;\;\;\;\;\;\;\;\;\;\;\;\;\;\;\;+\beta l3nrhd_{it}\times I\left(\gamma_{2}<\ln rhd_{it}\leq \gamma_{3}\right)+C+\varepsilon_{it} \end{array} \end{array}$$

where *Y* represents the domestic large cycle level of region *i* in year *t*; *X* is the control variable including environmental governance, industrial structure, and urban form; and Ecoit is the threshold variable for regional high-quality development. Where γ is a fixed threshold value, *α* is the coefficient of influence of *X_it_* on the efficiency of collaborative innovation, *β_1_* and *β_2_*, the threshold variables Ecoit are *γ_1_* and Ecoit ≤, respectively, the coefficient of influence on medical collaborative innovation efficiency at >*γ_1_*, C is a constant term, ε it a random perturbation term, and *I* (·) is a schematic function. Similarly, the formula for the double-threshold test is as follows (*β_2_* and *β_3_* have a meaning similar to *β_1_*):

Through the threshold regression model in the Stata 15 software, the regional production, distribution, circulation, consumption, foreign investment attraction, and international trade level are taken as the core explanatory variables. The single-threshold assumption and the double-threshold assumption are tested, and the test results are shown in Table 7. It can be seen that the results of the single-threshold test are more significant than those of the double-threshold test, so the results of the single-threshold regression were selected for further analysis. Thresholds 1 and 2 for each core explanatory variable were both 0.830 and 3.140, but there were still minor differences in the confidence intervals for each threshold. The results of the threshold test show that the regional healthcare development level is greater than 0.83 and less than 0.83, which will lead to different effects of domestic and international cycles on the efficiency of collaborative innovation.

**Table 7:** Threshold regression test results.

	lnproduce	lnincome	lntransport	lnconsume	lnfdi	lntrade
Single threshold	39.710***	34.060***	41.300***	47.370***	47.810***	38.71***
Double threshold	8.440	6.670*	6.930	14.320	13.340	7.630
Threshold value 1	0.830	0.830	0.830	0.830	0.830	0.830
Threshold value 2	3.140	3.140	3.140	3.140	3.140	3.140
Confidence interval 1	[0.740,0.840]	[0.820,0.840]	[0.820,0.840]	[0.750,0.840]	[0.750,0.840]	[0.715,0.840]
Confidence interval 2	[2.885,3.385]	[2.885,3.300]	[2.885,3.300]	[2.885,3.300]	[2.885,3.300]	[2.885,3.385]

The data in the table are F statistics corresponding to the threshold test. ***, ** and * are significant at the level of 1, 5 and 10%, respectively.

Table 8 presents the regression results of the panel sill model. According to the threshold values of different regional healthcare development levels and the corresponding coefficients of the core explanatory variables, the impact of domestic and international economic cycles on the efficiency of collaborative innovation can be analyzed under the economic development levels of two different regions. Specifically, when the regional healthcare development level is [0, 0.830], 78.82% of the samples of the regional healthcare development level of each city in the past years are in this range, which is the scope of the largest impact of the domestic and international economic cycles on the efficiency of collaborative innovation. Among them, samples in the central region accounted for 40.91%, samples in the western region accounted for 33.34%, and samples in the eastern region accounted for 25.75%. In this range, compared with other factors, FDI stock has the most obvious role in promoting the efficiency of collaborative innovation, and for every unit increase in the logarithmic value of regional FDI stock, medical collaborative innovation efficiency will increase by 0.014 units accordingly. The coefficient of influence of the allocation level is second only to FDI stock, but only about one-third of the latter coefficient, indicating that for every 1 unit increase in the value of the allocation level, the efficiency of collaborative innovation will increase by 0.004 units. The remaining coefficients are positive, indicating that domestic and international economic cycles improve medical collaborative innovation efficiency. When the regional healthcare development level was 0.830 or above, 21.18% of the samples in the regional healthcare development level of each city in the calendar year were in this range, which is smaller than the sample range below the threshold value. Among them, the sample from the eastern, central, and western regions accounted for 72.68, 14.60, and 12.72%, respectively. In this range, the coefficients of influence of various parts of the domestic and international economic cycles on the efficiency of collaborative innovation did not change in the order of size, but the specific coefficient values increased to varying degrees.

**Table 8:** Coefficient estimation results of threshold regression model.

	lnproduce	lnincome	lntransport	lnconsume	lnfdi	lntrade
lnproduce		0.001 (0.001)	0.001 (0.001)	0.001 (0.001)	0.001 (0.001)	0.001 (0.001)
lnincome	0.004*** (0.001)		0.004*** (0.001)	0.004*** (0.001)	0.004*** (0.001)	0.004*** (0.001)
lntransport	0.001 (0.001)	0.001 (0.001)		0.001 (0.001)	0.001 (0.001)	0.001 (0.001)
lnconsume	0.003*** (0.001)	0.003*** (0.001)	0.003*** (0.001)		0.003*** (0.001)	0.003*** (0.001)
lnfdi	0.014*** (0.000)	0.014*** (0.001)	0.014*** (0.001)	0.014*** (0.000)		0.014*** (0.001)
lntrade	0.002*** (0.001)	0.002*** (0.001)	0.002*** (0.001)	0.002*** (0.001)	0.002*** (0.001)	
lnelectric	0.004*** (0.001)	0.004*** (0.002)	0.004*** (0.001)	0.004*** (0.001)	0.004*** (0.001)	0.004*** (0.002)
lnfixed	0.005*** (0.001)	0.005*** (0.002)	0.006*** (0.001)	0.006*** (0.001)	0.005*** (0.001)	0.005*** (0.001)
lnstructure	0.025*** (0.002)	0.025*** (0.006)	0.026*** (0.002)	0.025*** (0.003)	0.025*** (0.002)	0.025*** (0.003)
(lnrhd ≤ γ_1_)	0.001 (0.001)	0.004*** (0.001)	0.001 (0.001)	0.003*** (0.001)	0.014*** (0.001)	0.001*** (0.001)
(lnrhd > γ_1_)	0.001 (0.001)	0.006*** (0.001)	0.001 (0.001)	0.006*** (0.001)	0.019*** (0.001)	0.002*** (0.001)
R^2^	0.792	0.791	0.788	0.794	0.793	0.792

***, ** and * represent significance levels of 1, 5 and 10%, respectively.

Based on the analysis results, the impact of the level of economic development on the efficiency of collaborative innovation is positive in each link above and below the threshold value, but the specific coefficient size is different. After the regional healthcare development level crossed the threshold value of 0.830, the regression coefficients of the core explanatory variables in the regression increased significantly. When the allocation level is used as the core explanatory variable, the elasticity coefficient of the allocation level to the efficiency of collaborative innovation increases from 0.004 to 0.006 after crossing the threshold value, while the consumption level, the level of attracting foreign investment, and the level of international trade are used as the core explanatory variables. Similarly, the elastic coefficient of medical collaborative innovation efficiency also increased from 0.003, 0.014, and 0.001 to 0.006, 0.019, and 0.002, respectively, after crossing the threshold. When the production level and flow level are the core explanatory variables, the regression coefficients of the core explanatory variables above and below the threshold value are not significant, and there is no significant size change. Overall, the domestic and international economic cycles in regions with higher levels of regional healthcare development have a more obvious role in promoting the efficiency of collaborative innovation, which may stem from the closer relationship between regional industry-university-research collaborative innovation and regional healthcare development with a better level of regional development, while the domestic and international economic cycles reflect regional healthcare development from the perspective of specific links.

## 5 Conclusion and policy recommendations

Starting from the perspective of the dual circulation strategy, this study uses the relevant statistics of 283 cities in China from 2003 to 2018 to use the GS2SLS spatial econometric model to view the real circulation strategy of the new development pattern. The spatial spillover effect of medical collaborative innovation efficiency improvement is empirically analyzed, and the threshold effect of “domestic and international economic cycles “on medical collaborative innovation efficiency is further explored. The results of the study show that: (1) overall, after considering the spatial spillover effect of medical collaborative innovation efficiency and controlling endogeneity, there is a significant spatial positive correlation between the regional healthcare development level and medical collaborative innovation efficiency. (2) From the perspective of the regional dual circulation strategy, the role of attracting foreign investment in the regional economic international cycle is the most obvious, and the role played by the domestic economic cycle has been significantly improved after the launch of the innovation-driven strategy. (3) The results of the threshold regression show that the factors of the dual circulation strategy will show different degrees of promotion effect on the efficiency of collaborative innovation above and below the threshold value of 0.83 in the regional healthcare development level, and the promotion effect under the higher regional healthcare development level is more obvious. Combined with the analysis results, this study proposes the following policy suggestions:

First, from the perspective of the dual circulation strategy, the development of the regional economy is necessary to promote innovative development in China. The development of innovative activities must be supported by a strong regional economy, and innovation without economic activities will become a source of water and wood. Therefore, it is not possible to separate regional healthcare development and innovation-driven development strategy, and it should be rationally planned and implemented for the development of local innovation undertakings according to local conditions. However, it is necessary to be aware of the significant spatial spillover effect of innovation, the role of innovation leadership in central cities, and the need to strengthen innovation cooperation and collaboration among various regions to better achieve the balanced development of innovation in various regions.

Second, from the empirical results, since the introduction of the innovation-driven strategy, the improvement of the domestic economic cycle level has played an increasingly important role in promoting the efficiency of collaborative innovation, therefore, under the new development pattern of “dual circulation strategy,” the regional healthcare development idea of taking the domestic economic cycle as the main body and participating in the international cycle will help promote the improvement of medical collaborative innovation efficiency. With the improvement in China’s economic development level, economic growth will be more dependent on endogenous innovation inputs, and the dependence on the introduction of innovation will be relatively weakened. Against the background of the new era and the pattern of economic development, all aspects of domestic economic development are of great significance to innovation, therefore, it is necessary to better grasp and make good use of the positive externalities brought about by regional healthcare development. China’s economic development has entered a new stage of speed reduction and quality improvement, and independent innovation and development are not only urgently needed for China’s economic development and industrial transformation and upgrading. Moreover, its effects are bound to inevitably appear in China after decades of rapid development and the construction of the entire industrial chain system to the current stage. However, China should always adhere to the principle of open and inclusive development, improve the quality and efficiency of opening up, and continue to promote a high level of opening up to further play an increasingly active role in global economic governance and actively participate in the international economic cycle.

Third, there are still huge differences in the level of development between various regions in China, and the practical problems and development directions faced under the new development pattern will inevitably be different. It is necessary to consider the implementation of differentiated policies to promote the improvement of medical collaborative innovation efficiency. The eastern region, which has better economic development, must adhere to high-quality development, achieve a high level of openness, take the initiative to participate in the international economic cycle, and give full play to the leading role of national economic development and innovation. The central region, which is in a state of rapid development and a period of industrial transformation, should continue to pay attention to the follow-up construction of various institutional guarantees while maintaining economic growth, and avoiding imbalance and inadequacy of development, so that the broadest masses of people can effectively enjoy the dividends brought by regional healthcare development, so as to promote the improvement of local medical collaborative innovation efficiency through all-round and inclusive economic performance. The western region, which is still in an underdeveloped state, must recognize its shortcomings in terms of regional healthcare development and innovation, focus on improving the local economic development level and the living standards of residents, actively promote the implementation of the rural revitalization strategy, and consolidate the fruits of the battle against poverty to compensate for the shortcomings and deficiencies in the development of local people’s livelihood while ensuring the local economic development level, so as to better achieve innovative development.

## Data availability statement

Publicly available datasets were analyzed in this study. This data can be found at: https://www.stats.gov.cn/sj/zxfb/index.html.

## Author contributions

LC: Data curation, Formal analysis, Investigation, Methodology, Writing – original draft, Writing – review & editing. JH: Conceptualization, Data curation, Formal analysis, Methodology, Writing – review & editing. XG: Data curation, Formal analysis, Methodology, Writing – original draft, Writing – review & editing.

## Footnotes

^1^There are 115 cities in the eastern region, including 12 provinces, autonomous regions and municipalities directly under the central government in Hebei, Liaoning, Jiangsu, Zhejiang, Fujian, Shandong, Guangdong, Guangxi, Hainan, Beijing, Tianjin and Shanghai; there are 109 cities in the central region, including nine provinces and autonomous regions in Shanxi, Jilin, Heilongjiang, Anhui, Jiangxi, Henan, Hubei, Hunan and Inner Mongolia; and 59 cities in the western region, including Sichuan, Guizhou, Yunnan, Shaanxi, Gansu, Ningxia, Qinghai, and Xinjiang, eight provinces, and autonomous regions.
